# Transformation of Terpenoids and Steroids Using Actinomycetes of the Genus *Rhodococcus*

**DOI:** 10.3390/molecules29143378

**Published:** 2024-07-18

**Authors:** Polina Yu. Maltseva, Natalia A. Plotnitskaya, Irina B. Ivshina

**Affiliations:** 1 Institute of Ecology and Genetics of Microorganisms of the Ural Branch of the Russian Academy of Sciences, Perm Federal Research Center of the Ural Branch of the Russian Academy of Sciences, 13 Golev Str., 614081 Perm, Russia; inbox.98@bk.ru (P.Y.M.); luchnikova.n@mail.ru (N.A.P.); 2 Department of Microbiology and Immunology, Perm State University, 15 Bukirev Str., 614990 Perm, Russia

**Keywords:** actinomycetes, biotransformation, steroids, terpenoids, *Rhodococcus*

## Abstract

Terpenoids and steroids are secondary plant and animal metabolites and are widely used to produce highly effective pharmacologically significant compounds. One of the promising approaches to the transformation of these compounds to form bioactive metabolites is their transformation using microorganisms. *Rhodococcus* spp. are one of the most developed objects in biotechnology due to their exceptional metabolic capabilities and resistance to extreme environmental conditions. In this review, information on the processes of biotransformation of terpenoid and steroid compounds by actinomycetes of the genus *Rhodococcus* and their molecular genetic bases are most fully collected and analyzed for the first time. Examples of the use of both native whole-cell catalysts and mutant strains and purified enzyme systems for the production of derivatives of terpenoids and steroids are given.

## 1. Introduction

Actinomycetes are a large group of Gram-positive bacteria that are widespread in terrestrial and aquatic ecosystems. The phylum *Actinomycetota* is represented by six valid classes: *Actinomycetes*, *Acidimicrobiia*, *Coriobacteriia*, *Nitriliruptoria*, *Rubrobacteria*, and *Thermoleophilia* (https://lpsn.dsmz.de/phylum/actinomycetota, assessed on 18 June 2024). Among actinomycetes, there are various phenotypes, such as anaerobes, aerobes, spore-forming, unicellular, and filamentous forms. They have a relatively large genome, often exceeding 5 Mb, and are characterized by a high (from 50 to more than 70 mol. %) GC content in DNA. Morphology of different actinomycetal species varies from coccoid (e.g., *Micrococcus*) and rod-shaped (e.g., *Arthrobacter*) forms to fragmented (e.g., *Nocardia*) or highly differentiated branched mycelium (e.g., *Streptomyces*). Actinomycetes exist in a free-living form or as commensals or symbionts of other organisms [1]. They play an important role in the decomposition of organic substances such as cellulose and chitin and take part in the natural carbon cycle [2].

Representatives of *Actinomycetes* produce a large number of biotechnologically significant enzymes, such as amylases, cellulases, proteases, chitinases, xylanases, peroxidases, nitrile hydratases, and pectinases [3]. More than 22,000 biologically active microbial metabolites are known, 45% of which are catalyzed by actinomycetes [4].

One of the groups of microorganisms widely developed in biotechnology is actinomycetes of the genus *Rhodococcus sensu stricto*, characterized by a wide variety of transformable, hard-to-reach compounds [2,5,6]. The non-mycelial growth, the ability to produce biosurfactants, the polytrophy, and the lability of metabolic systems of rhodococci determine the expediency of their using in biocatalysis and fine synthesis [7,8,9,10,11]. A number of review articles have been published regarding the use of the biotechnological potential of rhodococci for the degradation and transformation of complex organic compounds [5,6,12,13,14,15,16]. At the same time, this prepared review is the first work on the summarizing and analysis of data on the use of actinomycetes only of the genus *Rhodococcus* for the transformation of terpenoid and steroid compounds.

Terpenoids and steroids are secondary metabolites of plants and animals with a wide range of biological activity. These compounds are actively used as scaffolds for the synthesis of new substances with high pharmacological potential. Steroids and their derivatives are widely used in medical practice [17,18], while terpenoids and their derivatives are just gaining popularity. At the moment, only a few terpene compounds have been used in clinical trials [19,20,21]. One of the most common ways to obtain valuable derivatives of terpenoids and steroids is by chemical modification. This usually requires high temperature and pH, the use of expensive reagents, and the introduction of protective groups of molecule reactive centers. Microbial transformation of terpenoids and steroids allows biologically active derivatives with high regio- and stereoselectivity under normal and environmentally friendly conditions to be obtained [10,22,23]. This review is an attempt to highlight the role of actinomycetes of the genus *Rhodococcus* as biotransformers of terpenoid and steroid compounds and show directions of research development in this field.

## 2. Using the Biochemical Potential of Whole Bacterial Cells

The use of native bacterial cells in biocatalysis is economically more profitable in comparison with the use of transforming enzyme systems or individual enzymes. Despite the exceptional selectivity and substrate specificity of individual enzymes, their use is limited by low stability and a narrow range of metabolizable substrates. The range of substances that can be transformed by whole bacterial cells is much wider. Their use allows complex transformations to be carried out in one technological stage with a high degree of selectivity and in environmentally friendly conditions. In addition, bacteria secrete all necessary cofactors and provide a natural environment for enzymes, preventing conformational changes in the structure of proteins and loss of their reactivity [24]. Thus, the specificity of multi-purpose enzyme systems, the absence of problems with the regeneration of coenzymes, and stable activity in extreme environmental conditions make the use of living cells of rhodococci in biocatalysis technologically promising.

### 2.1. Transformation of Terpene Compounds

Terpenes are widespread and structurally diverse unsaturated hydrocarbons that are derivatives of mevalonic acid, whose structure is based on isoprene units (C_5_H_8_). Terpenes are the largest class of natural compounds and have more than 50,000 known representative molecules [25].

**Figure 1:**
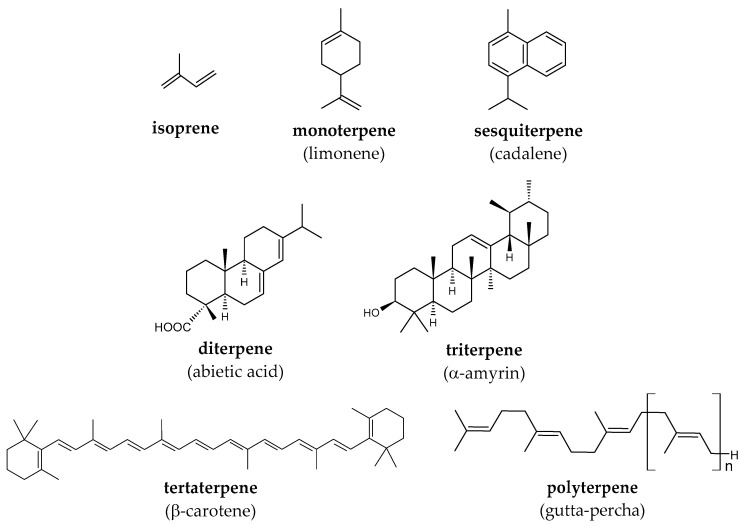
Representatives of terpenes.

Oxygen-containing terpenes are called terpenoids [26]. They are secondary metabolites of plants and often highly hydrophobic. According to the number of isoprene units in the structure of the molecule, there are mono-, sesqui-, di-, tri-, tetra-, and polyterpenoids (Figure 1).

In plants, terpenoids and their derivatives perform a variety of functions, acting as photosynthetic pigments, electron carriers, and regulators of growth and development. In addition, these compounds are involved in protection against insects, pathogens, and extreme environmental factors [27]. Terpenoids have a wide range of biological activity, exhibiting anti-inflammatory, antimicrobial, antiviral, analgesic, antitumor, antifungal, and other actions [25,28]. It is also worth noting that in recent years there has been a growing interest in plant terpenoids as a promising base for the production of biofuels [29]. In this regard, there is an active development of modern ways of using terpenoids and their derivatives in various fields of industry. One of the promising directions is their microbial transformation, aimed at obtaining biologically active derivatives. This approach makes it possible to eliminate the difficulties associated with the use of native substrates, and at the same time carry out transformation reactions according to the principles of “green chemistry” and the strategy of sustainable development [30].

At the moment, a large amount of knowledge has been accumulated about biotransformations of mono-, di-, and triterpene compounds using bacteria, fungi, and even insect larvae. The vast majority of the described studies on bioconversion by actinomycetes of the genus *Rhodococcus* are devoted to the transformation of monoterpene substrates such as limonene, carveol, 1,8-cineol, etc. Only in recent years, has a tendency to study biotransformations of more complex cyclic terpene compounds been traced.

**Figure 2:**
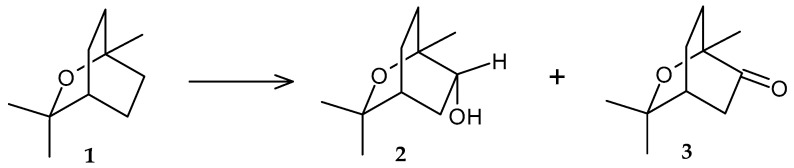
Scheme of biotransformation of 1,8-cineol (**1**) by *Rhodococcus* sp. C1 cells [31].

The first information about the catalytic activity of *Rhodococcus* spp. in relation to terpenoids was obtained in the late 1980s. Already at that time, much attention was paid not only to the technological process of conversion and its optimization but also to the features of microorganism enzyme systems involved in the studied transformations. Williams et al. (1989) reported that the isolated strain *Rhodococcus* sp. C1 utilizing 1,8-cineole (**1**) as the only carbon source accumulated 6-*endo*-hydroxycineol (**2**) and 6-oxocyneol (**3**) (Figure 2) [31]. Subsequently, the obtained metabolites, together with the initial substrate, were oxidized by washed bacterial cells. The studies revealed that 6-*endo*-hydroxycineol dehydrogenase and NADH-dependent 6-oxocineol oxygenase were presumably involved in the conversion.

**Figure 3:**
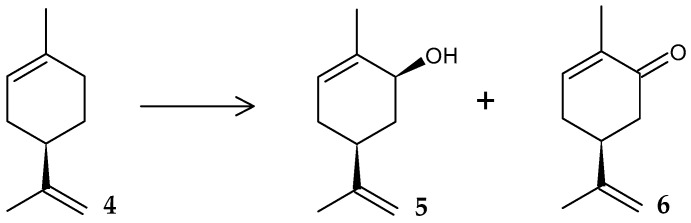
Scheme of biotransformation of limonene (**4**) by *R*. *opacus* PWD4 cells [32].

It was shown that *R. opacus* PWD4 is capable of conducting toluene-induced enantioselective hydroxylation of *D*-limonene (**4**) to form (+)-*trans*-carveol (up to 97%) (**5**) and small amounts of (+)-carvone (**6**) (Figure 3). It was suggested that toluene-degrading enzymes may participate in the catalysis, since the change of the co-substrate from toluene to glucose led to a loss in the transformation activity of rhodococci in relation to limonene [32].

An extensive series of studies carried out by various scientific groups is devoted to the catalytic activity of *R. erythropolis* DCL14 against monoterpenoids limonene-1,2-epoxide, carveol, and limonene. The first data on this strain as an effective biotransformer of (+)-limonene were presented by van der Werf and de Bont in 1998 [33]. The intermediate products obtained during degradation, limonene oxide and *p*-menth-7-ene-l,2-diol, did not represent great biotechnological value; however, they enabled an expansion of the understanding of the pathways of microbial metabolism of limonene and created a foundation for future research.

**Figure 4:**
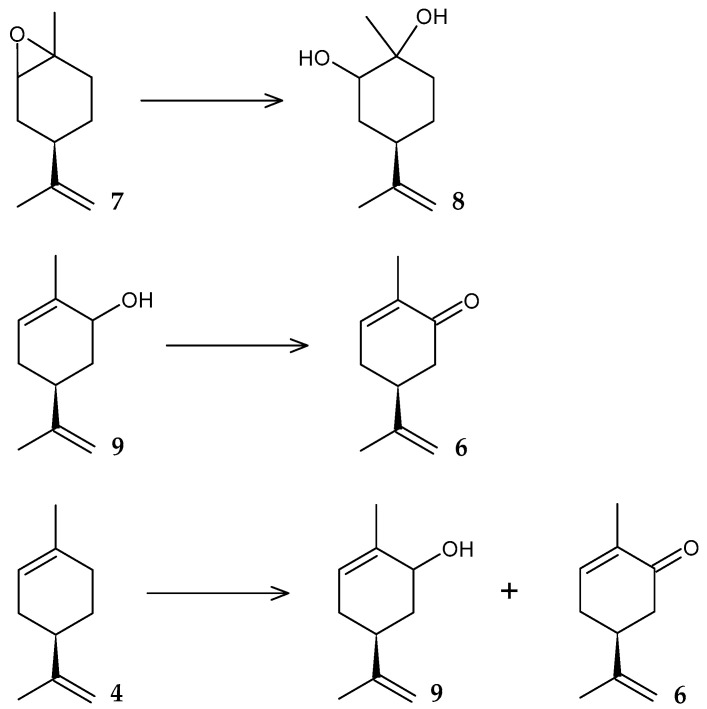
Scheme of biotransformations of limonene-1,2-epoxide (**7**), (−)-carveol (**9**), and limonene (**4**) by *R. erythropolis* DCL14 cells [34,35,36].

Subsequently, a research group led by Carla C.C.R. de Carvalho (Portugal) selected optimal conditions for the use of native *R. erythropolis* DCL14 cells. Thus, the inclusion of an organic solvent containing the substrate in the reaction system made it possible to increase the rate of hydrolysis of limonene-1,2-epoxide (**7**) to limonene-1,2-diol (**8**), oxidation of (−)-carveol (**9**) to carvone (**6**), and conversion of limonene (**4**) to carveol (**9**) and carvone (**6**) (Figure 4) [34,35,36]. Fractions of essential oils of *Citrus* and *Cymbopogon* plants contained carvone, *cis*-carveol, and *trans*-carveol possess antibacterial, antioxidant, and antiproliferative properties [37,38].

**Figure 5:**
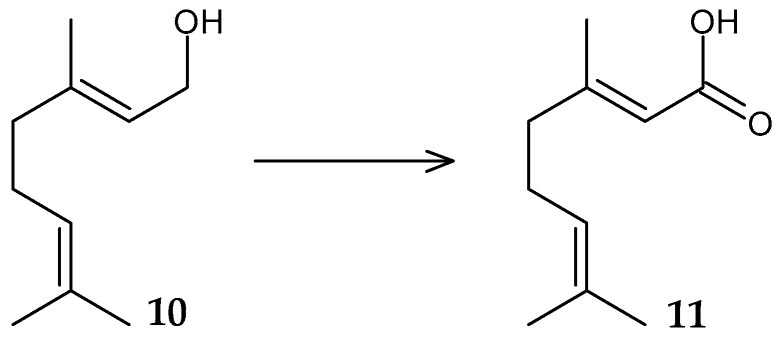
Scheme of biotransformation of geraniol (**10**) by *Rhodococcus* sp. GR2 cells [39].

A strain of *Rhodococcus* sp. GR3 isolated from soil carried out the oxidation of geraniol (**10**) to geranic acid (**11**) with a yield of up to 54.6% (Figure 5) [39]. Geranic acid has tyrosinase inhibitory activity [40] and is used for the synthesis of esters, with pronounced insecticidal activity against aphids [41].

**Figure 6:**
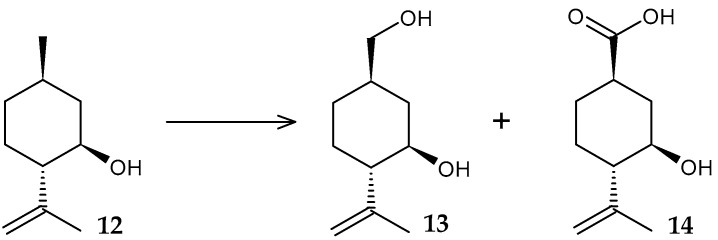
Scheme of biotransformation of (−)-isopulegol (**12**) by *R*. *rhodochrous* IEGM 1362 cells [11].

We previously studied the bioconversion of monoterpene alcohol (−)-isopulegol (**12**) by *R. rhodochrous* IEGM 1362 cells with the formation of two new 10-hydroxy (**13**) and 10-carboxy (**14**) derivatives (Figure 6) [11]. According to the results of the *in silico* analysis, the obtained metabolites may have antitumor activity, and are also promising as respiratory analeptics and agents for the prevention of cancer of the genitourinary system.

**Figure 7:**
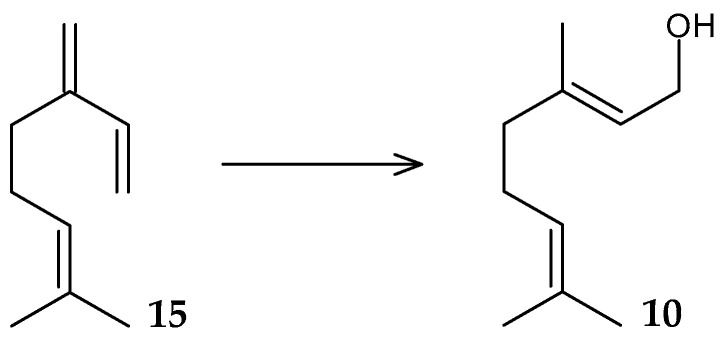
Scheme of biotransformation of β-myrcene (**15**) by resting cells of *R*. *erythropolis* MLT1 [42].

The ability of resting cells of *R. erythropolis* MLT1 to transform the acyclic monoterpene β-myrcene (**15**) was revealed (Figure 7) [42]. On the basis of this substrate being used as the only carbon source, the monoterpene alcohol geraniol (**10**), which has antibacterial activity against clinical strains of staphylococci and enterobacteria [43] and is widely used in perfumery, the food industry, and as an insect repellent [44], was obtained.

In recent years, in addition to monoterpene substrates, the ability of *Rhodococcus* actinomycetes to convert more complex organic compounds such as diterpene resin acids and lupane and oleanane pentacyclic triterpenoids has been revealed [45,46,47]. Interestingly, of the rather large species diversity of cultures used in screening studies, representatives of *R. rhodochrous* showed the greatest catalytic activity. At the same time, the greatest efficiency of microbial conversion was achieved using resting rhodococcal cells (washed from the medium at the end of the exponential growth phase and placed in a buffer with a substrate) [47]. Being in this state, bacterial cells have an increased energy resource specifically for biotransformation of the substrate. In addition to increasing the amount of the resulting transformation product, this approach allows for more precise control of the amount of biomass and also does not require sterile transformation conditions.

**Figure 8:**
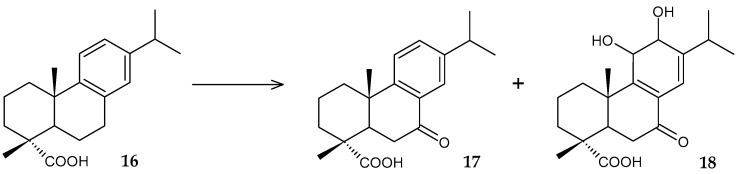
Scheme of biotransformation of dehydroabietic acid (**16**) by resting cells of *R. rhodochrous* IEGM 107 [45].

An effective biocatalyst of the biodegradation process of toxic dehydroabietic acid (**16**) has recently been developed using resting cells of *R. rhodochrous* IEGM 107 [45], and the formation of two less ecotoxic intermediates, 7-oxo-DHA (**17**) with moderate antibacterial activity [48] and 11,12-dihydroxy-7-oxo-abieta-8,13-dien-18-oic acid (**18**), has been detected (Figure 8).

**Figure 9:**
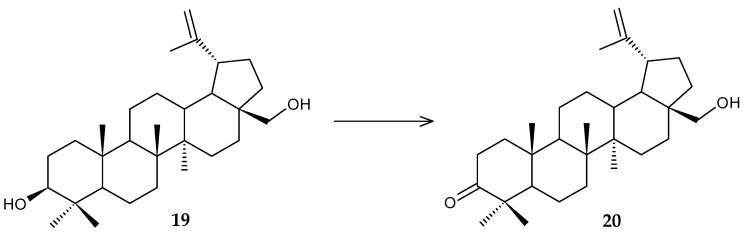
Scheme of biotransformation of betulin (**19**) by *R*. *rhodochrous* IEGM 66 cells [46].

The regioselective oxidation of the 3β-hydroxyl group of the pentacyclic triterpenoid betulin (**19**) was studied using *R. rhodochrous* IEGM 66 cells (Figure 9) [46]. The metabolite betulone (**20**) obtained as a result of transformation is a promising intermediate in the synthesis of cytotoxic derivatives [49]. The highest yield of the product (up to 75%) was achieved using resting actinomycete cells, while the maximum catalytic activity of growing cells was only 45%.

**Figure 10:**
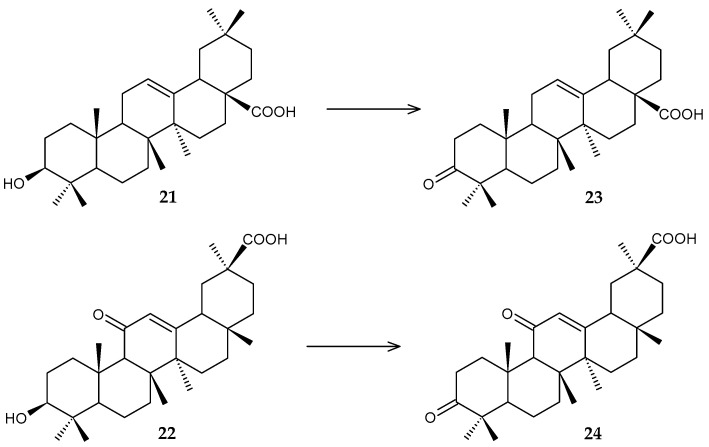
Scheme of biotransformations of oleanolic (**21**) and glycyrretinic (**22**) acids by *R. rhodochrous* IEGM 1360 cells [47].

Resting cells of *R. rhodochrous* IEGM 1360 showed high catalytic activity against oleanolic (**21**) and glycyrretinic (**22**) acids with the formation of 3-oxo derivatives (**23**, **24**) (with an efficiency of 61% and 100%, respectively) (Figure 10) [47]. The use of a suspension of resting cells in comparison with the transformation in growth conditions allowed the conversion time to be reduced from 7 to 3 days. 3-Oxo derivatives of oleanolic and glycyrrhetinic acids are known as anti-inflammatory [50,51], cytotoxic [52,53,54], antiparasitic [55,56], and antiviral [57] agents.

**Figure 11:**
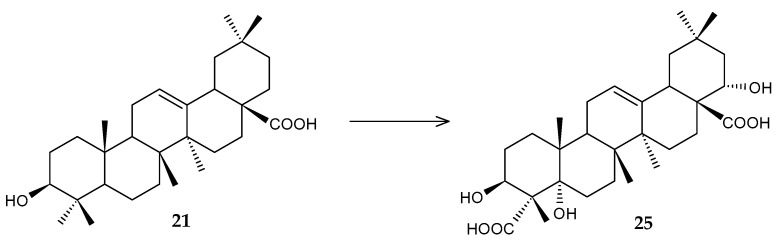
Scheme of biotransformation of oleanolic acid (**21**) by *R*. *rhodochrous* IEGM 757 cells [58].

More complex transformations of oleanolic acid (**21**) were achieved using *R. rhodochrous* IEGM 757 [58]. As a result of the complete conversion of the initial substrate, a new bioactive metabolite, 3β,5α,22α-trihydroxyolean-12-ene-23,28-dioic acid (**25**), was obtained (Figure 11).

### 2.2. Transformation of Steroid Compounds

Steroids and sterols are biogenetically close to terpenoids and are formed from triterpene precursors. Steroids and sterols are compounds of animal or plant origin that are widespread in nature and have high biological activity. They are components of cell membranes, act as signaling molecules, and also play an important role in the development of cancer.

**Figure 12:**
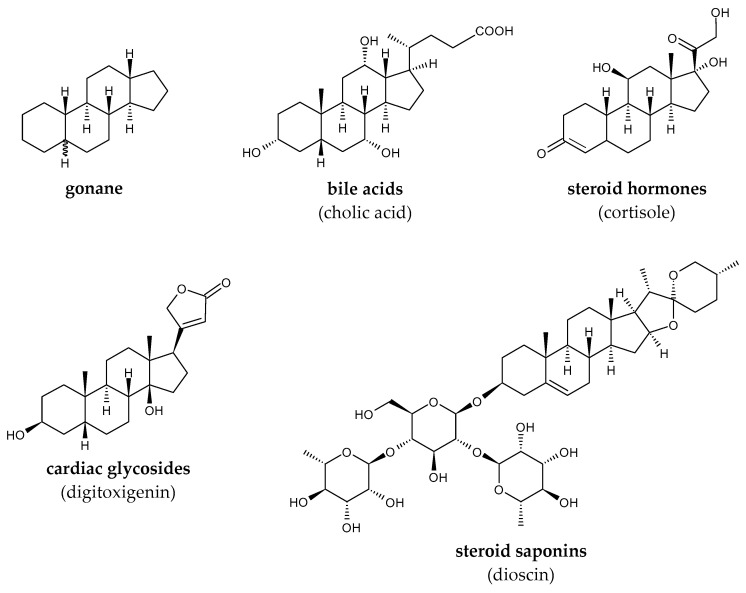
Representatives of steroids.

The basis of the steroid skeleton is cyclopentane perhydrophenanthrene (sterane, gonane), the structure of which consists of four carbon rings, three cyclohexane and one cyclopentane. Based on biological function or activity, steroids can be classified into several types: bile acids, steroid hormones, cardioactive glycosides and aglycones, and steroid saponins (Figure 12) [59].

Native steroid compounds, when used as medicines, often cause various side effects. Modification of the side chain of steroid molecules makes it possible to obtain compounds with more pronounced biological activity, which helps to reduce drug dosages and, as a result, reduce the manifestation of undesirable side effects.

There are many steroid compounds among environmental pollutants, which accumulate in reservoirs and exhibit negative environmental effects. In this regard, another area of microbial transformations of steroids is their degradation in order to purify the environment from pharmaceutical pollutants [60]. Having a relatively high hydrophobicity, steroids do not biodegrade easily. Nevertheless, the mechanisms of steroid metabolism using aerobic (including *Rhodococcus*) and anaerobic microorganisms, as well as the key stages and enzymes involved in the process of steroid conversion and genes encoding them, have been studied in detail [61]. Of particular interest to researchers are methods of obtaining pharmacologically significant steroid derivatives based on the use of genetic engineering of *Rhodococcus* spp. cells.

**Figure 13:**
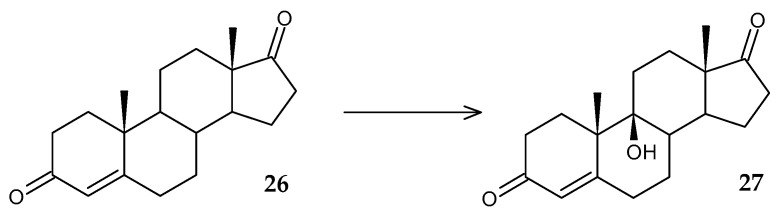
Scheme of biotransformation of AD (**26**) by *Rhodococcus* sp. cells [62].

9α-Hydroxy derivatives of steroids are valuable intermediates in the synthesis of compounds with targeted anti-inflammatory activity. A large part of studies on the hydroxylation of androst-4-ene-3,17-dione (AD) by actinomycetes of the genus *Rhodococcus* was carried out by a group of scientists from Bulgaria [62]. The difficulty of this reaction lies in the high activity of Δ^1^-steroid dehydrogenase of microorganisms. When this enzyme is combined with 9α-steroid hydroxylase, an unstable intermediate 9α-hydroxy-l,4-androstadiene-3,17-dione is formed, and subsequently complete degradation of the initial substrate occurs. For the first time, the ability of rhodococci to direct biotransformation of AD (**26**) into 9α-hydroxy-4-androstene-3,17-dione (9α-OH-AD) (**27**) was recorded using *Rhodococcus* sp. isolated from oil-contaminated soil (Figure 13) [62]. However, the yield of the product when using the substrate in a concentration of no more than 3 g/L reached only 70%.

The subsequent series of research concerned the optimization of the process of 9α-hydroxylation of AD using *Rhodococcus* sp. IOC-77 cells by the selection of optimal substrate solvents, the composition and characteristics of the reaction medium, and the effect on the enzyme systems of microorganisms [63,64,65,66,67]. The most significant results were achieved in experiments with blocking protein synthesis and preliminary adaptation of bacterial cells to the substrate, which allowed for the complete conversion of AD to 9α-OH-AD [64]. Recently, data have been obtained on the use of rhodococci pre-grown on *n*-alkanes to double the yield of 9α-OH-AD compared with the efficiency of using cells grown on glucose [67].

The strain *R. erythropolis* Ac-1740 obtained as a result of directed selection showed the ability to completely convert AD to 9α-OH-AD [68]. The advantage of this strain is its low degradation activity against the steroid core due to the absence of 3-ketosteroid-1,2-dehydrogenase, which greatly simplifies the selection of optimal reaction conditions and the further use of a biocatalytic system for the production of the target product. Thus, a method has been developed for the production of 9α-OH-AD from a mixture of soy sterols using the microbial association *Mycobacterium neoaurum* Ac-1634 and *R. erythropolis* Ac-1740. According to Andryushina et al. [69], *M. neoaurum* Ac-1634 cells transformed the initial mixture of phytosterols (sitosterol, stigmasterol, campesterol, and saturated sterols) into AD, the subsequent 9α-hydroxylation of which to the target product occurred with the participation of *R. erythropolis* Ac-1740 cells. It should be noted that the use of these biocatalysts for the first time made it possible to increase the initial concentration of the substrate to 13.5 g/L.

**Figure 14:**
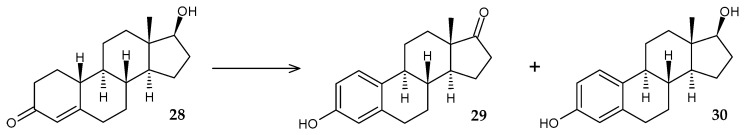
Scheme of biotransformation of 19-nortestosterone (**28**) by *Rhodococcus* sp. DSM 92-344 cells [70].

In addition to 9α-hydroxylation, another reaction catalyzed by rhodococci is aromatization of the A ring of steroid molecules. A group of scientists from Egypt [70] discovered the ability of *Rhodococcus* sp. DSM 92-344 to aromatize 19-nortestosterone (**28**) to form estrone (**29**) and estradiol (**30**) (Figure 14), with a maximum overall conversion efficiency of up to 77%. Further optimization of the steroid biotransformation process using an air-lift column allowed for an increase in the yield of products to 56 and 23%, respectively [71].

**Figure 15:**
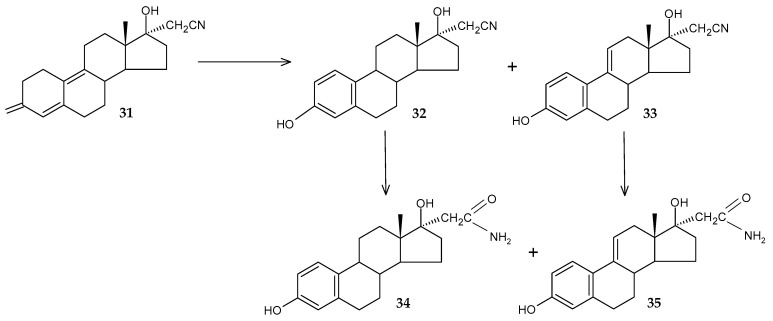
Scheme of biotransformation of dienogest (**31**) by *R*. *erythropolis* FZB 53 cells [72].

The transformation of progestin dienogest (**31**) using *R. erythropolis* FZB 53 cells was studied in order to obtain pharmaceutically active compounds [72]. Despite the authors’ supposed manifestation of nitrile hydrolase activity by this strain, the main direction of dienogest transformation was the aromatization of the A ring with the formation of estra-1,3,5(10)-triene (**32**) and 1,3,5(10),9(11)-tetraene (**33**) compounds, which subsequently transformed to 17α-acetamide derivatives of estradiol (**34**) and 9(11)-dehydroestradiol (**35**) (Figure 15). However, the process of obtaining target products **34** and **35** turned out to be quite long and was up to 27 days.

**Figure 16:**
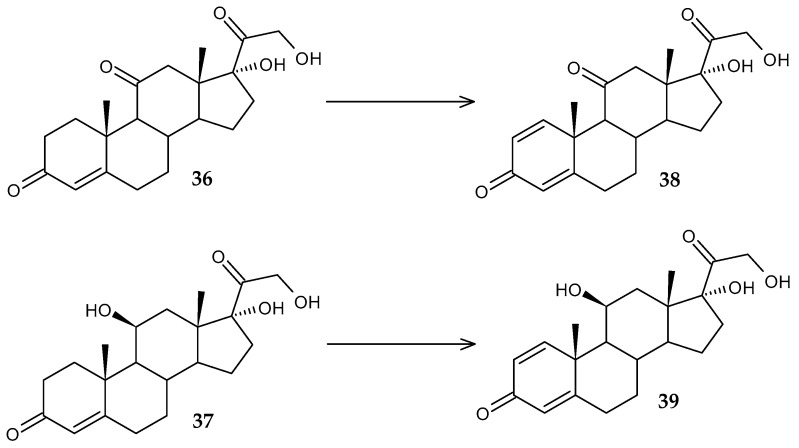
Scheme of biotransformation of cortisone (**36**) and hydrocortisone (**37**) by *R*. *coprophilus* DSM 43347 cells [73].

Costa et al. [73] conducted research on the development of an alternative method for the synthesis of therapeutically effective steroids prednisone and prednisolone. The glucocorticoids cortisone and hydrocortisone have anti-inflammatory activity, but their use is difficult due to many side effects. In this regard, it is promising to use more effective derivatives of these compounds, prednisone and prednisolone, which allows for lower doses of the drug. According to the results of screening 13 rhodococcal strains, *R. coprophilus* DSM 43347 showed the highest target activity, catalyzing Δ^1^-dehydrogenation of cortisone (**36**) and hydrocortisone (**37**), with the formation of prednisone (**38**) (94%) and prednisolone (**39**) (97%), respectively (Figure 16).

**Figure 17:**
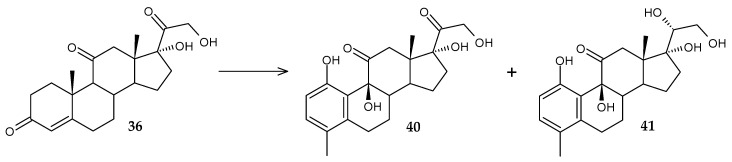
Scheme of biotransformation of cortisone (**36**) by *R. rhodnii* DSM 43960 cells [74].

Based on cortisone (**36**), new compounds with potential cytotoxic activity, 1,9β,17,21-tetrahydroxy-4-methyl-19-nor-9β-pregna-1,3,5(10)-trien-11,20-dione (70%) (**40**) and 1,9β,17,20β,21-pentahydroxy-4-methyl-19-nor-9β-pregna-1,3,5(10)-trien-11-one (20%) (**41**), were obtained using whole *R. rhodnii* DSM 43960 cells (Figure 17) [74]. Additionally, the time of complete conversion of the initial substrate was only 24 h.

**Figure 18:**
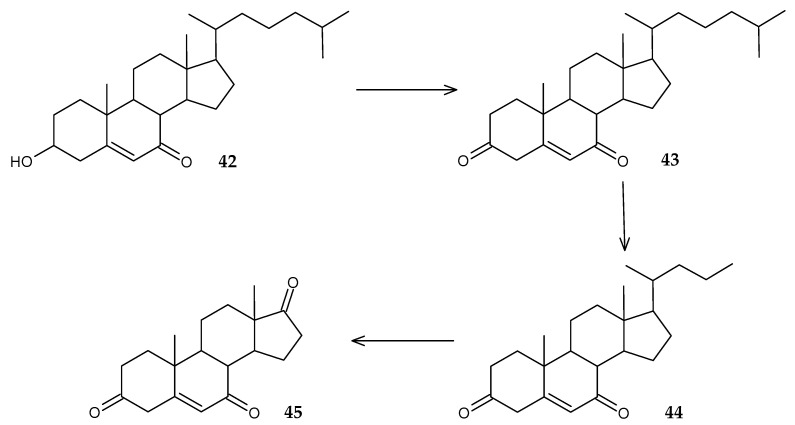
Scheme of biotransformation of 7-ketocholesterol (**42**) by *R*. *erythropolis* MTCC 3951 cells [75].

It has been shown that *R. erythropolis* MTCC 3951 is a promising strain for the biodegradation of 7-ketocholesterol (**42**), which has a cytotoxic effect and causes various age-related pathologies [75]. As a result of optimization, 93% degradation of the substrate was achieved and the participation of the enzymes cholesterol oxidase, lipase, dehydrogenase, and reductase in the process of biodegradation of 7-ketocholesterol (**42**) was revealed. 4-Cholesten-3,7-dione (**43**), chol-5-en-3,7-dione (**44**), and androsta-4-ene-3,7,17-trione (**45**) were identified as intermediates (Figure 18).

Hao et al. (2024) obtained interesting data on *Rhodococcus* sp. RSBS9 isolated from dairy farm soil capable of efficient degradation of 17β-estradiol under low-temperature conditions [76]. The strain retained catabolic activity against the steroid at 10 °C (up to 94%) and even 5 °C (56%). The obtained information indicates the prospects of using rhodococci to develop methods for the treatment of polluted ecosystems at low temperatures.

## 3. Using Bacterial Enzymes

The ability of bacterial cells to transform various compounds is due to the action of their enzyme systems. A logical trend in the development of biotechnology is the isolation and purification of bacterial enzymes, as well as their overexpression in model organisms in order to scale the production of target compounds. The use of individual enzymes allows for highly selective reactions with repeated use, reduces sterility requirements, and simplifies the process of isolation and purification of the target product [24]. In this regard, studies of the catalytic activity of *Rhodococcus* spp. in relation to terpene and steroid substrates were accompanied by the study of microbial enzymes involved in transformations, as well as functional genes encoding them.

### 3.1. Transformation of Terpene Compounds

At the end of the last century, Dutch microbiologists obtained data about a new pathway for the biodegradation of (4*R*)- and (4*S*)-isomers of limonene by *R. erythropolis* DCL14 cells [77,78]. The enzymes limonene-1,2-monooxygenase, limonene-1,2-epoxide hydrolase, limonene-1,2-diol dehydrogenase, and 1-hydroxy-2-oxo-limonene-1,2-monooxygenase participated in these transformations of limonene. In addition, the *limA* gene encoding limonene-1,2-epoxide hydrolase was identified and overexpressed in *E. coli* [79].

The same research group isolated and purified a new enzyme from *R. erythropolis* DCL14, nicotinoprotein dichlorophenolindophenol-dependent carveol dehydrogenase, which catalyzes the oxidation of carveol to carvone (see Figure 4), and revealed limonene and carveol-induced specific action of this enzyme [80].

The *cinA1* gene localized in the *CinTMP1* operon and encoding cytochrome P450 was identified in the genome of *R. josii* TMP1, a biotransformer of 1,8-cineol [81]. Using recombinant *E. coli* and *R. erythropolis* cells, it was found that this enzyme catalyzes the oxidation of 1,8-cineol to 6-oxocineol (see Figure 2).

**Figure 19:**
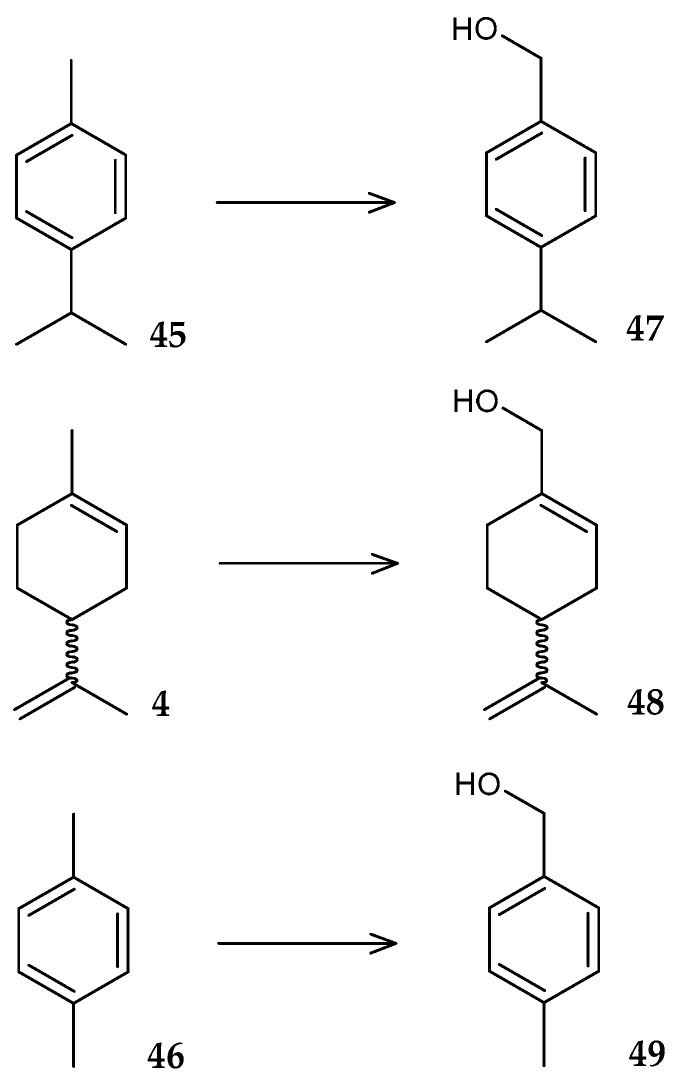
Scheme of biotransformations of *p*-cymene (**45**), (*R*/*S*)-limonene (**4**), and *p*-xylene (**46**) using CYP102N12 [82].

The strain *R. globerulus* JDV-SF1993 isolated from the rhizosphere of *Eucalyptus* sp. contains genes of the enzyme CYP102N12, belonging to the cytochrome P450 family [82]. It was found that CYP108N12 catalyzes the region-specific hydroxylation of *p*-cymene (**45**), (*R*/*S*)-limonene (**4**), and *p*-xylene (**46**) to 4-isopropylbenzyl alcohol (**47**), perillyl alcohol (**48**), and *p*-tolylmethanol (**49**), respectively (Figure 19).

**Figure 20:**
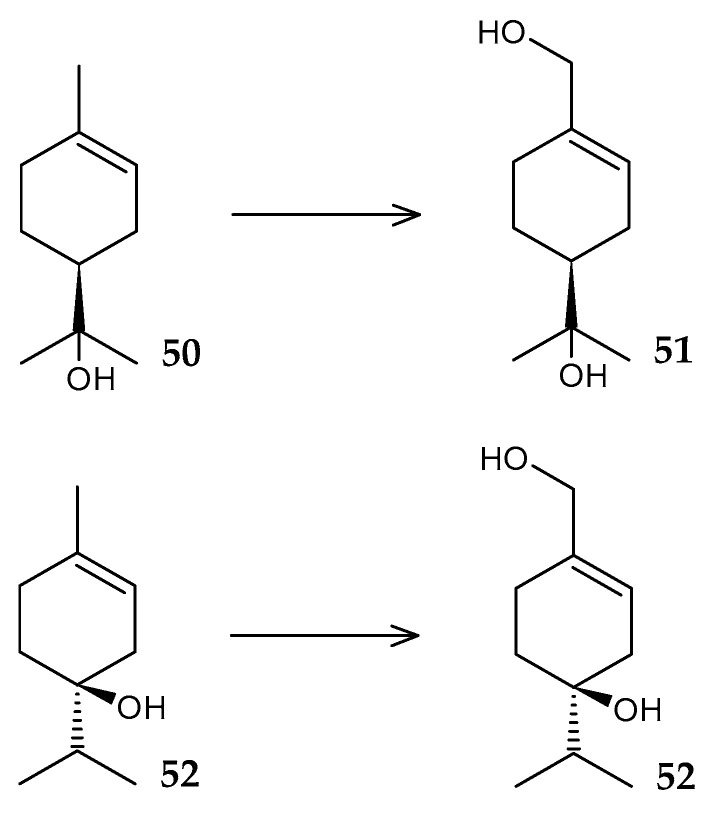
Scheme of biotransformations of (*S*)-α-terpineol (**50**) and (*S*)-4-terpineol (**52**) using CYP102N14 [83].

Subsequent studies of the genome of *R. globerulus* JDV-SF1993 revealed a gene encoding CYP108N14 [83]. This enzyme, like the previously described CYP102N12, hydroxylates *p*-cymene (**45**) and (*R*/*S*)-limonene (**4**), and also transforms (*S*)-α-terpineol (**50**) to (*S*)-7-hydroxyterpineol (**51**) and (*S*)-4-terpineol (**52**) to (*S*)-7-hydroxy-4-terpineol (**53**) (Figure 20).

### 3.2. Transformation of Steroid Compounds

Currently, there is a tendency to isolate, analyze, and heterologously express enzymes that catalyze the reactions of the microbial transformation of steroids. This approach allows you to expand the range of target products and scale their production in the future [84].

**Figure 21:**
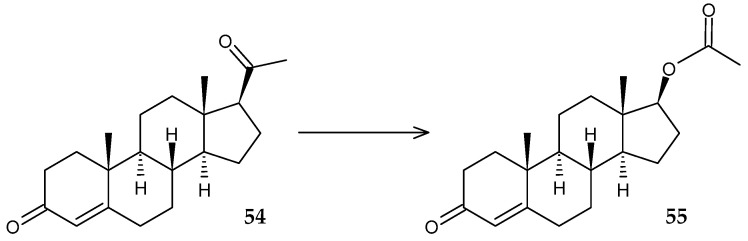
Scheme of biotransformation of progesterone (**54**) using *R*. *rhodochrous* monooxygenase [85].

Steroid NADPH-dependent monooxygenase isolated from *R. rhodochrous* cells catalyzes the oxidation of progesterone (**54**) through the Bayer–Villiger reaction to form testosterone acetate (**55**) (Figure 21) [85].

**Figure 22:**
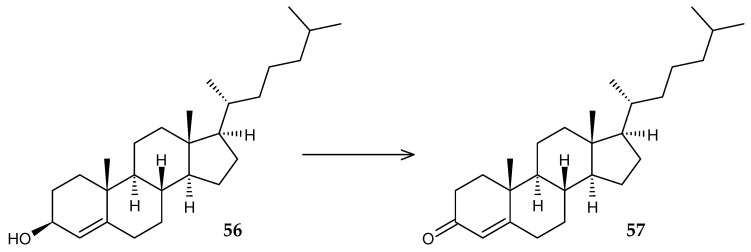
Scheme of biotransformation of cholesterol (**56**) using cholesterol oxidase.

Many representatives of *Rhodococcus* sp. are characterized by the presence of extracellular cholesterol oxidase, which catalyzes the conversion of 3β-hydroxy-Δ^5^-sterols into the corresponding 3-keto-Δ^4^-derivatives (Figure 22). The *ChoG* gene corresponding to this enzyme is found in strains belonging to the species *R. erythropolis* [86], *R. ruber* [87], *R. triatomae* [88], and *Rhodococcus* sp. [89], and is often located next to the genes of 3-ketosteroid-∆^1^-dehydrogenase (*KstD*) and 3-ketosteroid-9α-hydroxylase (*KshAB*).

The regulation and transcription mechanisms of various isoforms of the *KshAB* gene are of particular interest to researchers. Based on data obtained by Baldanta et al. (2021), it appears that isoforms KshA2 and KshA3 are the primary enzymes involved in the degradation of AD and cholesterol, respectively, while KshA1 has a supporting role in these processes [90].

Using a mutant strain derived from *Rhodococcus* sp. MIL 1038, a new compound identified as 7aβ-methyl-1β-[1,5-dimethyl-6-hydroxyl-hexyl]-5-oxo-3aα-hexa-4-indanepropionic acid was obtained from cholesterol. The resulting metabolite can be used as an intermediate for the chemical synthesis of steroids with pharmaceutical potential [91].

It was revealed that *R. erythropolis* SQ1 contains two isoforms of 3-ketosteroid-1-dehydrogenase [92]. Complete suppression of the activity of this enzyme by deletion of the *KstD* functional gene and UV mutagenesis made it possible to block the formation of by-products and subsequent degradation of the steroid skeleton. As a result, the resulting mutant strain *R. erythropolis* RG1-UV29 catalyzed the reaction of effective transformation of AD to the target product 9-OH-AD, with a yield of 93%.

**Figure 23:**
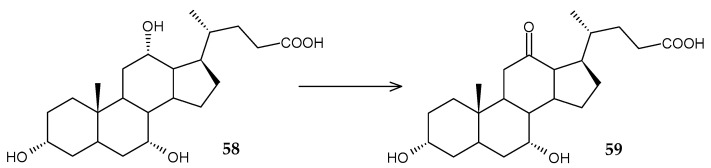
Scheme of biotransformation of cholic acid (**58**) using (NAD^+^)-dependent 12α-hydroxysteroid dehydrogenase [93].

A new (NAD^+^)-dependent 12α-hydroxysteroid dehydrogenase (*Rr*12α-HSDH) has been identified in the genomic sequence of *R*. *ruber* [93]. The purified enzyme demonstrated high catalytic activity against cholic acid (**58**), carrying out its oxidation to 12-oxochenodeoxycholic acid (**59**) (85%) (Figure 23).

**Figure 24:**
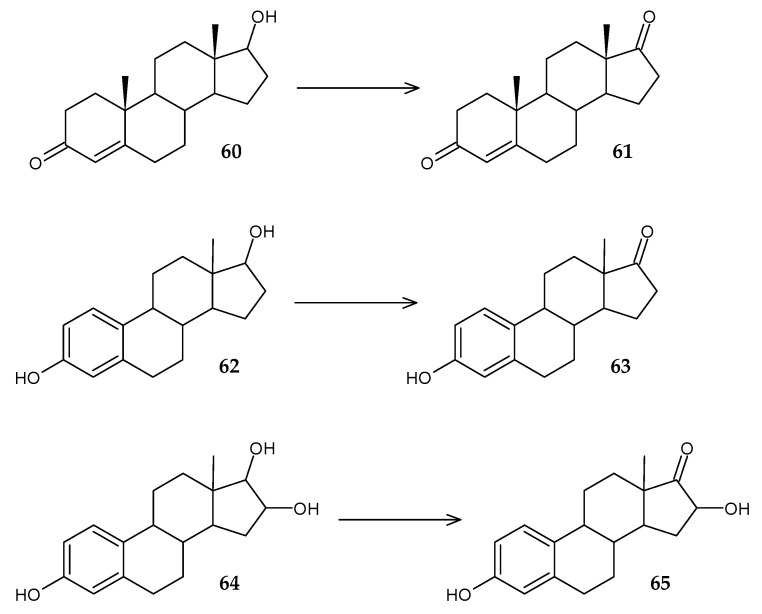
Scheme of biotransformations of estradiol (**60**), estriol (**62**), and testosterone (**64**) using 17β-hydroxysteroid dehydrogenase [94,95].

It is known that the initial stage of microbial degradation of steroids is dehydrogenation in the C17 position, which determines the prospects of using steroid dehydrogenases for bioremediation. A gene encoding 17β-hydroxysteroid dehydrogenase (17β-HSD) was identified in the genome of *Rhodococcus* sp. P14 [94]. Recombinant *E. coli* BL21 cells expressing this enzyme were able to transform estradiol (**60**) into estrone (**61**) with an efficiency of up to 94%. Later, the same research group showed that *Rhodococcus* sp. P14 also uses other steroids, including estriol and testosterone, as a sole carbon source [95]. Genome screening made it possible to identify the gene for short-chain dehydrogenase (17β-HSDx), which catalyzes the conversion of estradiol (**60**) to estrone (**61**), estriol (**62**) to 16-hydroxyestrone (**63**), and testosterone (**64**) to androst-4-en-3,17-dione (**65**) (Figure 24).

**Figure 25:**
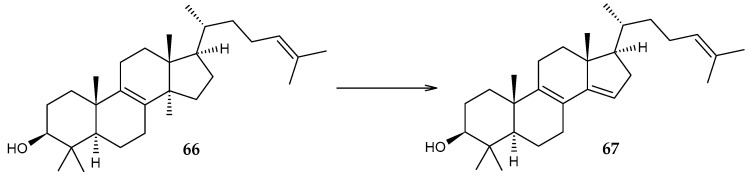
Scheme of biotransformation of lanosterol (**66**) using CYP51 [96].

The 14α-demethylase CYP51 of *R. triatomae* BKS 15-14, which catalyzes the demethylation of lanosterol (**66**), has been identified and characterized (Figure 25) [96].

**Figure 26:**
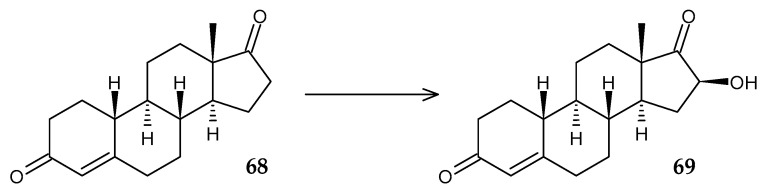
Scheme of biotransformation of norandrostenedione (**68**) by mutant cells of *R*. *erythropolis* RG9 [97].

Rhodococci can be used not only as sources of steroid transformation enzymes but also as effective recipients for heterologous expression of functional genes. Thus, the *R. erythropolis* RG9 strain containing the P450 BM3 mutant M02 enzyme from *Bacillus megaterium* transformed 17-ketosteroid norandrostenedione (**68**) into 16β-OH norandrostenedione (**69**), with a product yield of more than 95% (Figure 26) [97].

**Figure 27:**
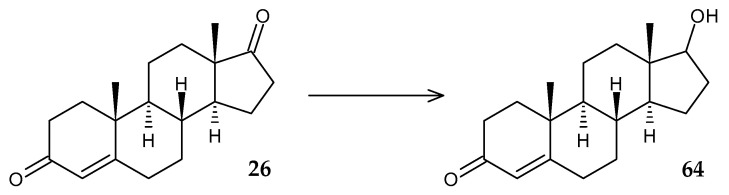
Scheme of biotransformation of AD (**26**) by mutant cells of *R*. *ruber* Chol-4 [98].

*R. ruber* Chol-4 was used as a model organism for overexpression of the 17-ketoreductase gene (*17β-hsd*) of the pathogenic fungus *Cochliobolus lunatus* in order to obtain testosterone (**64**) from AD (**26**) (Figure 27) [98]. In addition, the genome of the resulting mutant strain was characterized by the deletion of four genes (*KshB*, *KstD1*,*2*,*3*), which prevented degradation of the substrate. Despite the low yield of the target product (61%) and the rather complex process of mutagenesis, *R. ruber* Chol-4 is of interest for further research, since the overwhelming amount of testosterone was secreted extracellularly.

**Figure 28:**
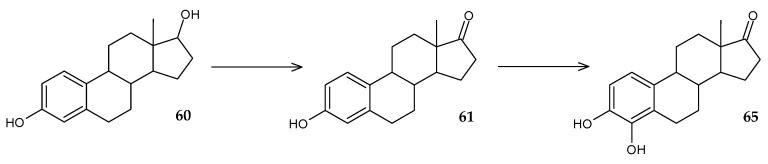
Scheme of biotransformation of 17β-estradiol (**60**) by *R*. *equi* DSSKP-R-001 cells [99].

Recently, the 4,5-seco pathway of biodegradation of 17β-estradiol by *R*. *equi* DSSKP-R-001 was discovered, and enzymes and their encoding genes involved in the initial stages of catabolism were identified [99]. It was found that the initial 17β-estradiol (**60**) is dehydrogenated by short-chain dehydrogenase (*hsd17b14* gene) to estrone (**61**), which, in turn, is transformed by flavin-binding monooxygenase (*At1g12200*) to form 4-hydroxyestrone (**65**) (Figure 28). The subsequent cleavage of the steroid ring A is catalyzed by 3-hydroxy-9,10-secoandrosta-1,3,5(10)-triene-9,17-dion monooxygenase (*hsaC*) and catechol-1,2-dioxygenase (*catA*).

## 4. Conclusions

Taking into account the historical development of the discussed topic and focusing on data from recent years, we conducted an extensive analysis of information on the use of actinomycetes of the genus *Rhodococcus* for the transformation of terpenoid and steroid compounds. It was shown that the biotransformation of terpenoid compounds is accompanied, as a rule, by hydroxylation, carboxylation, and dehydration reactions, whereas the conversion of steroids proceeds along more complex pathways, with the reactions of aromatization of the steroid ring and the introduction of double bonds and amide groups. Currently obtained derivatives of terpenoids and steroids are known for their anti-inflammatory, antibacterial, antioxidant, antitumor, insecticidal, and antiviral activity, etc. [25,28,100]. Analysis of *Rhodococcus* species used as biotransformers revealed that *R*. *erythropolis*, *R*. *globerulus,* and *R*. *rhodochrous* possess the highest catalytic activity (Table 1).

Among the advantages of using whole-cell catalysts, it may be noted that they are cheap and stable, and there is no need to add additional cofactors for enzyme systems. At the same time, such catalysts as a rule have limitations on the maximum concentration of the transformed starting substrate (usually up to 5 g/L) and the rate of transformation, often require additional sources of carbon (such as toluene, succinate, ethanol, yeast extract, etc.), and also carry out undesirable side reactions [11,31,32,45,70,72,74] due to the redundancy of metabolic systems, which leads to contamination of the product and an increase in its final cost. The tendency to use purified enzyme systems for the directed conversion of terpenoid and steroid substrates makes it possible to obtain valuable metabolites with a high level of purity in a short amount of time but requires additional costs for the purification and stabilization of such systems.

The rhodococcal genes and enzyme systems involved in the processes of steroid transformation were studied in detail. Data on the genes and enzymes responsible for the transformation of terpene compounds are scarce. Among the identified enzyme systems of rhodococci involved in the conversion of terpenes and steroids, enzymes of the families of hydrolases, hydroxylases, dehydrogenases, demethylases, oxidases, and oxygenases, including CYP450, were identified.

**Figure 29:**
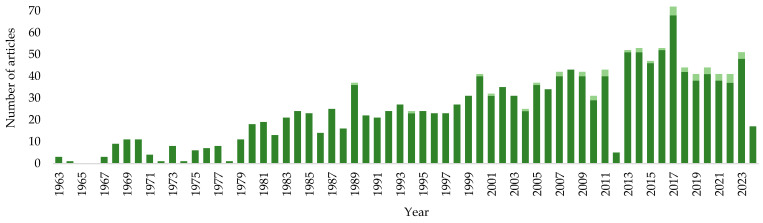
Number of articles devoted to the biotransformation of terpenoids and steroids (according to PubMed). Light green indicates the number of articles where *Rhodococcus* actinomycetes were used.

The data obtained indicate the pronounced biocatalytic potential of actinomycetes of the genus *Rhodococcus* and the prospects for further research aimed at studying the features and molecular genetic basis of the biotransformation processes of natural compounds of the terpenoid and steroid groups. According to the PubMed service, since the middle of the 20th century, the number of articles devoted to the biotransformation of terpenoids and steroids has grown (Figure 29). Though *Rhodococcus* actinomycetes started to be actively used for terpenoid and steroid biotransformation in the beginning of the 21st century (Figure 29, light green), research in this field is expected to significantly expand.

## Figures and Tables

**Table 1:** Biotransformation of terpenoids and steroids by actinomycetes of the genus *Rhodococcus*.

Substrate	Strain	Type of Catalyst	Type of Reaction	Derivatives	Reference
Terpenoids					
1,8-Cineole	*Rhodococcus* sp. C1	Native cells	Hydroxylation, oxidation	6-*endo*-Hydroxycineol, 6-oxocyneol	[31]
	*R. josii* TMP1	Enzyme P450_cin_ (gene *cinA1*)	Oxidation	6-Oxocineol	[81]
Limonene	*R. opacus* PWD4	Native cells	Hydroxylation, oxidation	(+)-*trans*-Carveol, (+)-carvone	[32,36]
	*R. erythropolis* DCL14	Native cells	Oxidation	Limonene oxide, *p*-menth-7-ene-l,2-diol	[33]
	*R. erythropolis* DCL14	Enzymes limonene 1,2-monooxygenase, limonene-1,2-epoxide hydrolase	Oxidation, epoxidation	Limonene-1,2-diol	[77,78]
	*R. globerulus* JDV-SF1993	Enzymes CYP108N12, CYP108N14	Hydroxylation	Perillyl alcohol	[82,83]
Limonene- 1,2-epoxide	*R. erythropolis* DCL14	Native cells	Hydrolysis	Limonene-1,2-diol	[34]
		Enzyme limonene-1,2-epoxide hydrolase (gene *limA*)	Hydrolysis	Limonene-1,2-diol, hydroxy-2-oxolimonene, 3-isopropenyl-6-oxoheptanoate	[79]
Carveol	*R. erythropolis* DCL14	Native cells	Oxidation	Carvone	[35]
		Enzyme carveol dehydrogenase - CDH (gene *limC*)	Oxidation	Carvone	[80]
Geraniol	*Rhodococcus* sp. GR3	Native cells	Oxidation	Geranic acid	[39]
(−)-Isopulegol	*R. rhodochrous* IEGM 1362	Native cells	Hydroxylation, carboxylation	10-Hydroxy-(−)-isopulegol, 10-carboxy-(−)-isopulegol	[11]
β-Myrcene	*R. erythropolis* MLT1	Resting cells	Dehydrogenation	Geraniol	[42]
*p*-Cymene	*R. globerulus* JDV-SF1993	Enzymes CYP108N12, CYP108N14	Hydroxylation	4-Isopropylbenzyl alcohol	[82,83]
*p*-Xylene	*R. globerulus* JDV-SF1993	Enzyme CYP108N12	Hydroxylation	*p*-Tolylmethanol	[82]
(*S*)-α-Terpineol	*R. globerulus* JDV-SF1993	Enzyme CYP108N14	Hydroxylation	(*S*)-7-Hydroxyterpineol	[83]
(*S*)-4-Terpineol	*R. globerulus* JDV-SF1993	Enzyme CYP108N14	Hydroxylation	(*S*)-7-Hydroxy-4-terpineol	[83]
Dehydroabietic acid	*R. rhodochrous* IEGM 107	Resting cells	Oxidation, hydroxylation	7-Oxo-dehydroabietic acid, 11,12-dihydroxy-7-oxo-abieta-8,13-dien-18-oic acid	[45]
Betulin	*R. rhodochrous* IEGM 66	Native cells, resting cells	Oxidation	Betulone	[46]
Oleanolic acid	*R. rhodochrous* IEGM 1360	Resting cells	Oxidation	3-Oxo-oleanolic acid	[47]
	*R. rhodochrous* IEGM 757	Native cells	Hydroxylation, carboxylation	3β,5α,22α-Trihydroxyolean-12-ene-23,28-dioic acid	[58]
Glycyrretinic acid	*R. rhodochrous* IEGM 1360	Resting cells	Oxidation	3-Oxo-glycyrretinic acid	[47]
Steroids					
Androst-4-ene- 3,17-dione	*Rhodococcus* sp.	Native cells	Hydroxylation	9α-Hydroxy-4-androstene-3,17-dione	[62]
	*Rhodococcus* sp. IOC-77				[63,64,65,66,67]
	*R. erythropolis* Ac-1740				[68,69]
	*R. erythropolis* RG1-UV29	Mutant strain (deletion of gene *KstD*, UV irradiation)	Hydroxylation	9α-Hydroxy-4-androstene-3,17-dione	[92]
	*R. ruber* Chol-4	Mutant strain (insertion of gene *17β-hsd*, deletion of genes *KshB*, *KstD1*,*2*,*3*)	Dehydrogenation	Testosterone	[88]
19-Nortestosterone	*Rhodococcus* sp. DSM 92-344	Native cells	Aromatization	Estrone, estradiol	[70,71]
Dienogest	*R. erythropolis* FZB 53	Native cells	Aromatization, amination	17α-Acetamide-estradiol, 17α-acetamide-9(11)-dehydroestradiol	[72]
Cortisone	*R. coprophilus* DSM 43347	Native cells	Dehydrogenation	Prednisone	[73]
	*R. rhodnii* DSM 43960	Native cells	Hydroxylation, methylation	1,9β,17,21-Tetrahydroxy-4-methyl-19-nor-9β-pregna-1,3,5(10)-trien-11,20-dione, 1,9β,17,20β,21-pentahydroxy-4-methyl-19-nor-9β-pregna-1,3,5(10)-trien-11-one	[74]
Hydrocortisone	*R. coprophilus* DSM 43347	Native cells	Dehydrogenation	Prednisolone	[73]
7-Ketocholesterol	*R. erythropolis* MTCC 3951	Native cells	Oxidation, dehydrogenation	4-Cholesten-3,7-dione, chol-5-en-3,7-dione, androsta-4-ene-3,7,17-trione	[75]
17β-Estradiol	*Rhodococcus* sp. RSBS9	Native cells	ND *	ND *	[76]
	*Rhodococcus* sp. P14	Enzyme 17β-hydroxysteroid dehydrogenase (gene *17β-HSD*)	Dehydrogenation	Estrone	[94]
	*R*. *equi* DSSKP-R-001	Enzyme short-chain dehydrogenase (gene *hsd17b14*), flavin-binding monooxygenase (gene *At1g12200*)	Dehydrogenation, hydroxylation	Estrone, 4-hydroxyestrone	[99]
Estriol	*Rhodococcus* sp. P14	Enzyme short-chain 17β-hydroxysteroid dehydrogenase (gene *17β-HSD*x)	Dehydrogenation	16-Hydroxyestrone	[94]
Testosterone	*Rhodococcus* sp. P14	Enzyme short-chain 17β-hydroxysteroid dehydrogenase (gene *17β-HSDx*)	Dehydrogenation	Androst-4-en-3,17-dione	[94]
Progesterone	*R.* *rhodochrous*	Enzyme steroid monooxygenase	Oxidation	Testosterone acetate	[85]
Cholesterol	*R. erythropolis*	Enzyme extracellular cholesterol oxidase (gene *ChoG*)	Oxidation	3-Keto-Δ^4^-cholesterol	[86]
	*R. ruber*				[87]
	*R. triatomae*				[88]
	*Rhodococcus* sp.				[89]
	*Rhodococcus* sp. MIL 1038	Mutant strain (biochemical mutagenesis by NTG)	Hydroxylation, oxidation, carboxylation, carbon cycle decomposition	7aβ-Methyl-1β-[1,5-dimethyl-6-hydroxyl-hexyl]-5-oxo-3aα-hexa-4-indanepropionic acid	[91]
Cholic acid	*R. ruber*	Enzyme 12α-hydroxysteroid dehydrogenase (gene *Rr12α-HSDH*)	Dehydrogenation	12-Oxochenodeoxycholic acid	[93]
Lanosterol	*R. triatomae* BKS 15-14	Enzyme CYP51 (gene *RtCYP51*)	Demethylation	14α-Dimethyllanosterol	[96]
Norandrostenedione	*R. erythropolis* RG9	Mutant strain (insertion of CYP450 BM3 mutant M02 enzyme genes)	Hydroxylation	16β-Hydroxy-norandrostenedione	[97]

* No data.

## Data Availability

Data sharing is not applicable to this article.
